# Complete mitochondrial genome of Japanese bigeye (*Pristigenys niphonia*): genome characterization and phylogenetic analysis

**DOI:** 10.1080/23802359.2016.1258339

**Published:** 2016-12-23

**Authors:** Bo Xu, Yingzhe Wang, Haizhu Zhou, Changlong Gou, Wenlong Dong, Yu Wang, Yunhang Gao, Hongxia Ma

**Affiliations:** a College of Animal Science and Technology, Jilin Agricultural University, Changchun, China;; b Agro-Biotechnology Research Institute, Jilin Academy of Agricultural Sciences, Gongzhuling, Jilin, China

**Keywords:** Priacanthidae, *Pristigenys niphonia*, mitochondrial genome

## Abstract

The Japanese bigeye (*Pristigenys niphonia*) is a species in the genus *Pristigenys* and in the family Priacanthidae. To understand the phylogenetic relationship of Japanese bigeye in teleost, we firstly determined the complete mitochondrial genome of Japanese bigeye. The entire mitochondrial genome of Japanese bigeye is 16,519bp in length, including 13 protein-coding genes and 2 ribosomal RNA genes (rRNA), 22 transfer RNA genes (tRNAs), and 2 main non-coding regions. The overall base composition is 24.9% of T, 30.6% of C, 27.7% of A, and 16.8% of G. The gene arrangement, base composition, and tRNA structures of the complete mitochondrial genome of Japanese bigeye is consistent with those of other teleost. The complete mitochondrial genome of Japanese bigeye was used to construct phylogenetic tree, which shows that Japanese bigeye is clustered with the fishes of the order Perciformes. We expect that the availability of mitochondrial genome of Japanese bigeye will facilitate the further investigations of the taxonomic resolution, biogeography, and molecular systematic.

The Priacanthidae, the bigeyes, are a family of 4 genus and 19 species of marine fishes. As one of the member of the family Priacanthidae, the Japanese bigeye (*Pristigenys niphonia*) is native to Western Pacific: Japan, East China Sea Shelf, Taiwan, South China Sea, Vietnam, Celebes, Australia and Indonesia (Iwatsuki et al. 2012). To research the taxonomic resolution and phylogenetic relationships of Japanese bigeye with other vertebrates, we sequenced the complete nucleotide sequence for the mitochondrial genome of Japanese bigeye. The samples were collected from the East China Sea (25.75°N, 123.47°E) .The specimen was preserved in 95% ethanol at herbarium which located Jilin Agricultural University (accession number:DK204).

Mitochondrial DNA was extracted and kept at −20 °C. The mitogenome of Japanese bigeye is a closed double-stranded circular molecule of 16,519 nucleotides (GenBank accession number: KX641477) and contains 13 protein-coding genes, 2 rRNA genes, 22 tRNA genes, the control region (CR), and the origin of the light-strand replication (O_L_). The base composition of Japanese bigeye is 24.9% of T, 30.6% of C, 27.7% of A, and 16.8% of G. The A + T (52.6%) content is higher than G + C (47.4%) content, which is similar to other fishes (Cheng et al. 2011a; Jin et al. 2012). Furthermore, the anti-G bias is ascertained in the third position of protein-coding genes, which brings Red bigeye in line with other vertebrate mitogenomes (Cheng et al. 2012a; Jin et al. 2013). The two ribosomal RNA genes 12S rRNA (954bp) and 16S rRNA (1703bp) are located on the heavy strand between *tRNA^Phe^* and *tRNA^Leu^* (UUR), and being separated by *tRNA^Val^* gene. The 13 protein-coding genes are encoded on heavy-strand except for *ND6*, which is encoded on the light-strand. All the protein-coding genes start ATG, except for *COI*, which used GTG as the initiation codon. The stop codon of four protein-coding genes (*ND1*, *COI*, *ATP8*, and *ND4L*) is TAA, while *ND6* end with TAG. The remaining protein-coding genes (*ND2*, *COII*, *ATP6*, *COIII*, *ND3*, *ND4*, *ND5,* and *Cytb*) have incomplete stop codon, either TA– or T––, which is common to vertebrate mitochondrial protein-coding genes (Xu et al. 2011; Cheng et al. 2012b; Liu et al. 2013). And, these incomplete termination codons are presumably complete as TAA via posttranscriptional polyadenylation (Ojala et al. 1981). The 22 tRNA genes, which contains two forms of tRNA^Ser^ (UCN and AGY) and tRNA^Leu^ (UUR and CUN), scatter throughout the genome and range from 67 to 75 bp in size and the gene arrangement is typically as in most vertebrates. Among the 22 tRNA genes, 14 are located on the heavy-strand and 8 on the light-strand. Three tRNA clusters (IQM, WANCY, and HSL) are well conserved in Japanese bigeye as in other vertebrate mitochondrial genomes (Wei et al. 2013). Some overlaps occur in protein-coding genes and tRNAs ranging from 1 to 10 bp, which is similar to most vertebrates. The control region, which is determined to be 837bp, located between *tRNA^Pro^* and *tRNA^Phe^* and this characterization is consistent with those of other teleost (Cheng et al. 2011b). Remarkably, the conserved motif 5′-GCCGG-3′, which is involved in the transition from RNA to DNA synthesis (Thomson et al. 1986), was identified in the mitogenome of Japanese bigeye.

Phylogenetic analysis result based on the complete mitochondrial genome sequences demonstrated that Red bigeye is clustered with the fishes of the order Perciformes (Figure 1). We expect that this mitogenome sequence data would play a crucial role in phylogenetic analysis of Red bigeye.

**Figure 1.**
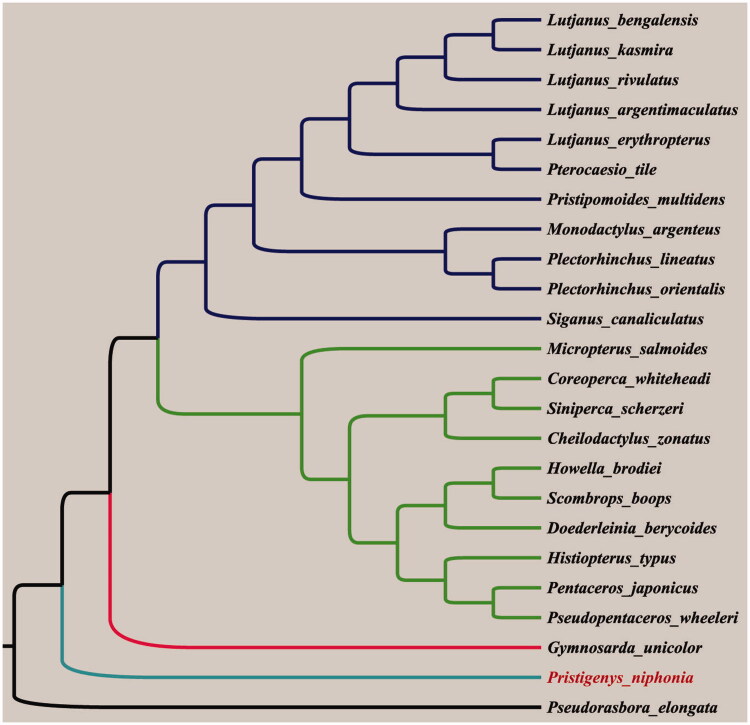
Phylogenetic tree was constructed using the neighbour-joining method by MEGA 5 based on the complete mitochondrial genome sequences. The mitochondrial genome sequences used in phylogenetic analyses were derived from GenBank, accession no.: *Cheilodactylus zonatus* AP006013.1, *Coreoperca whiteheadi* KJ149811.1, *Doederleinia berycoides* AP009181.1, *Gymnosarda unicolor* AP012510.1, *Histiopterus typus* AP006807.1, *Howella brodiei* AP014536.1, *Lutjanus argentimaculatus* JN182927.1, *L. bengalensis* FJ171339.1, *L. erythropterus* KP939271.1, *L. kasmira* FJ416614.1, *L. rivulatus* AP006000.1, *Micropterus salmoides* DQ536425.1, *Monodactylus argenteus* AP009169.1, *Pentaceros japonicus* AB739063.1, *Plectorhinchus lineatus* KM099284.1, *P. orientalis* KP966562.1, *Pristipomoides multidens* KF430626.1, *Pseudopentaceros wheeleri* AB741956.1, *Pterocaesio tile* AP004447.1, *Scombrops boops* LC006297.2, *Siganus canaliculatus* KJ872545.1, *Siniperca scherzeri* KF746199.1.
